# Sensorimotor-Conceptual Integration in Free Walking Enhances Divergent Thinking for Young and Older Adults

**DOI:** 10.3389/fpsyg.2016.01580

**Published:** 2016-10-13

**Authors:** Chun-Yu Kuo, Yei-Yu Yeh

**Affiliations:** ^1^Department of Educational Psychology and Counseling, National Pingtung UniversityPingtung, Taiwan; ^2^Department of Psychology, National Taiwan UniversityTaipei, Taiwan

**Keywords:** creativity, divergent thinking, cognitive processes, embodied cognition, aging

## Abstract

Prior research has shown that free walking can enhance creative thinking. Nevertheless, it remains unclear whether bidirectional body-mind links are essential for the positive effect of free walking on creative thinking. Moreover, it is unknown whether the positive effect can be generalized to older adults. In Experiment 1, we replicated previous findings with two additional groups of young participants. Participants in the rectangular-walking condition walked along a rectangular path while generating unusual uses for chopsticks. Participants in the free-walking group walked freely as they wished, and participants in the free-generation condition generated unconstrained free paths while the participants in the random-experienced condition walked those paths. Only the free-walking group showed better performance in fluency, flexibility, and originality. In Experiment 2, two groups of older adults were randomly assigned to the free-walking and rectangular-walking conditions. The free-walking group showed better performance than the rectangular-walking group. Moreover, older adults in the free-walking group outperformed young adults in the rectangular-walking group in originality and performed comparably in fluency and flexibility. Bidirectional links between proprioceptive-motor kinematics and metaphorical abstract concepts can enhance divergent thinking for both young and older adults.

## Introduction

Creativity is essential for the advancement of humanity. Creativity is a core activity in arts, sciences, entrepreneurship, and innovation in the workplace. It has been demonstrated that people with higher levels of creativity possess greater occupational self-efficiency (Tierney et al., 1999), healthier psychological functioning (McCracken, 1991; Terr, 1992; Russ, 1998; Kin and Pope, 1999), subjective well-being and successful adaption to daily demands (Reiter-Palmon et al., 1998), and better interpersonal relations (Livingston, 1999). Creativity also contributes to successful aging by encouraging flexible problem-solving skills and promoting self-efficacy in older adults (Fisher and Specht, 1999). In the era of aging and aged societies, empowering older adults with skills geared toward successful aging significantly affects society.

Divergent thinking is a core process of creativity (Guilford, 1967). Divergent thinking entails exploring many alternative and original ideas that differ from the standard responses to a given goal. Once a problem is defined, divergent thinking is critical for generating innovative, novel, and useful ideas. Given its importance, numerous methods have been developed to enhance divergent thinking such as brainstorming, structured lectures and exercises, social modeling, behavioral modification, and individualized coaching. Meta-analysis studies showed that programs emphasizing the development of cognitive skills using realistic, domain-specific exercises showed improvements, with effect size (*r*) ranging from 0.02 to 0.22 (Scott et al., 2004). Adult education programs focusing on divergent thinking strategies showed a large effect (*r* = 0.35) on improving fluency, flexibility, and originality indicators of the Torrance Test of Creative Thinking (Tsai, 2014). The benefit arises from near transfer of applying the trained cognitive strategies to tasks that require divergent thinking.

Three intervention methods that do not share overlapping cognitive processes could produce far transfer on divergent thinking ability. Induction of episodic-specific retrieval processes improves divergent thinking with medium (*r* = 0.25) and large (*r* = 0.36) effects for young adults (Madore et al., 2015). The second approach is to enhance creativity through physical exercises such as running and dancing. Gondola and Tuckman (1985) first demonstrated the benefit of long-term physical exercise on creative performance on Alternative Uses Test (AUT) and Remote Consequences that measured originality. Creativity can be improved by long-term and acute physical exercise (Gondola, 1986); aerobic exercise enhances creativity (Gondola, 1987; Steinberg et al., 1997) and the effect could last for 2 h (Blanchette et al., 2005). The third method is to enhance divergent thinking through mild bodily movement (Leung et al., 2012; Slepian and Ambady, 2012; Oppezzo and Schwartz, 2014). Fluid movements produced by tracing lines demonstrated a medium-to-large effect size (*r* = 0.25, 0.38, 0.40, and 0.46 for remote association, category inclusion, originality, and fluency, respectively) compared with rigid movements (Slepian and Ambady, 2012). Walking on a treadmill produced significant improvements compared with sitting (Oppezzo and Schwartz, 2014), using the indicators of appropriate uses (*r* = 0.33) and the number of total ideas (*r* = 0.44) in the AUT (Guilford et al., 1978). Moreover, free walking promoting metaphorical ideas related to divergent thinking enhanced performance in two tasks that required generating novel captions for ambiguous pictures (*r* = 0.28) and generating possible objects from viewing Lego assemblages (*r* = 0.15) compared with walking along a rectangular path (Leung et al., 2012).

Episodic-specificity induction enhances divergent thinking by encouraging recombining ideas across various episodes (Madore et al., 2015). Bodily movements influence divergent thinking by activating metaphorical abstract concepts (Leung et al., 2012; Slepian and Ambady, 2012). The use of a comfortable and self-selected pace in walking on a treadmill (Oppezzo and Schwartz, 2014) may promote a metaphor of unconstrained and free thoughts to enhance divergent thinking. Walking outdoors at one’s natural pace (Oppezzo and Schwartz, 2014) may also trigger this abstract concept. The contrast between walking freely inside a rectangle and walking on a rectangular path promotes thinking outside the box (Leung et al., 2012), which entails unconstrained thinking.

If bodily movements encourage creative processes by metaphors (Leung et al., 2012; Slepian and Ambady, 2012), it remains unknown whether bidirectional links between bodily movements and metaphorical abstract concepts are essential for observing the benefits of movements on divergent thinking. According to the simulated sensorimotor metaphor (SSM) theory (Slepian and Ambady, 2014), bidirectional links relating concrete sensorimotor states and metaphorical abstract concepts are critical for a newly learned embodied metaphor to influence sensorimotor judgments. In that study, participants were primed with a novel metaphorical abstract concept that related time (past, present) and weight (heavy) by reading statements. Participants were then requested to estimate the physical weight, popularity, and age of a scientific book that had an old or a new cover. Only the participants who were physically given the book showed the effect of newly learned metaphors, judging the weight according to the primed condition. Participants who simply viewed a photograph of the book did not show the effect. The metaphorical concept must contain the sensorimotor representations (heavy), and the sensorimotor states (feel the weight) must link to the concept for a novel metaphor (e.g., past-heavy) to influence weight judgment. We hypothesize that bidirectional links are also essential for bodily movements to benefit divergent thinking.

The first goal of this study was to verify the hypothesis, adopting the free-walking conditions used in Leung et al.’s (2012) study. We chose walking because walking is easily implemented by people at any age, at any time, with little cost and with no equipment. More importantly, we can verify the hypothesis by examining whether metaphorical abstract (free, unconstrained) concepts or the experienced proprioceptive-motor kinematics (fluid movements) alone can enhance divergent thinking. If either element is effective, it can be beneficial for disabled elders who require assistance to move around. Experiment 1 examined the boundary condition for free walking to enhance divergent thinking. Given the results of Experiment 1, we investigated whether free walking also enhanced divergent thinking for older adults in Experiment 2.

## Experiment 1

The purpose of this experiment was to investigate whether the bidirectional links between sensorimotor and metaphorical abstract concepts are critical for observing the benefits of free walking on divergent thinking. Leung et al. (2012) showed that participants who walked freely inside a square box performed better on divergent thinking than participants who walked along a rectangular path around the square box. Participants in the rectangular-walking condition did not show better performance compared with participants in the sitting condition. The null effect of the rectangular-walking condition may have arisen from two factors as follows: the rectangular box induced the metaphorical concept of constraining one’s thoughts, and the experienced proprioceptive-motor kinematics is rigid from walking along lines with straight angles.

To verify the hypothesis, we added the following two conditions: free-generation and random-experienced groups. Participants in the free-generation condition generated free paths for the participants in the random-experienced condition to walk. In the former group, the metaphorical abstract concept was induced without the corresponding sensorimotor experiences. In the latter group, participants walked the random paths without self-initiated abstract concepts in the mind-body connection. Although participants in this group walked predetermined paths as participants in the rectangular-walking condition, these participants walked along random paths comprising many curves. By contrast, participants in the rectangular-walking condition walked along a path comprising angular corners. It has been shown that tracing curved lines enhanced creativity because of the fluid movements compared with tracing lines with rigid angles (Slepian and Ambady, 2012). If bidirectional links between proprioceptive-motor kinematics and metaphorical concepts related to creative processes are critical for producing the benefits of free walking on divergent thinking, we expected that only the participants in the free-walking condition would perform better compared to the rectangular-walking group. The rectangular-walking condition served as the control group because participants in this condition demonstrated a performance comparable to the sitting group (Leung et al., 2012) and participants in this condition also experienced walking movements.

### Method

#### Participants

This study was approved by the Research Ethics Office of the National Taiwan University. Sixty-four undergraduate students (24 males, 40 females, mean at 23.95 years of age with *SD* of 2.72) volunteered in the experiment for course credit or monetary rewards. The participants were randomly assigned to four different conditions (*n* = 16 in each group). All of the participants had normal or corrected-to-normal vision and were naïve regarding the purpose of the experiment.

#### Design and Tasks

We followed the method used in Leung et al.’s (2012) Experiment 2b. The experiment was a single factor between-subjects design, with the four conditions as the sole variable. To evaluate divergent thinking, we adopted an AUT that required the participants to generate as many unusual uses as possible for a common object: chopsticks. To assure that any group differences did not arise from individual characteristics, we also assessed creativity using a Creativity Assessment Packet (CAP; Williams, 1980) questionnaire and basic cognitive functions using a two-alternative forced choice (2AFC) task to measure processing speed and a “hearts and flowers” task (Davidson et al., 2006; Diamond et al., 2007) to assess cognitive flexibility.

##### CAP instrument

The original version of CAP is a 50-item questionnaire originally designed by Williams (1980) to measure creativity tendency. We used the Chinese version of CAP (Lin and Wang, 1994). Participants reported how often they believed they had experiences referenced by each item on a 3-point Likert scale from “almost always” to “almost never.” Higher averaged total scores purportedly reflect higher tendency in the four dimensions of creativity: adventure, curiosity, imagination, and challenge. The revised CAP had good 4-week test–retest reliability (*r* = 0.61–0.74) and was positively correlated with the Pennsylvania Assessment of Creative Tendency (Rookey, 1973) and Torrance Tests of Creative Thinking (Torrance, 1987). We excluded the items related to experiences in school.

##### 2AFC task

Participants were asked to judge each presented stimulus by pressing the appropriate response key. Each trial began with the presentation of a fixed point for 1,500 ms. A red circle or a green triangle was then presented in the center of the screen. The participants were required to judge which target was presented by pressing the left key of the mouse for one target and the right key for the other. The corresponding key was counterbalanced across participants. The participants were asked to respond to each target as quickly and as accurately as possible.

##### Hearts and flowers task

Participants were asked to follow different stimulus-response mapping rules in different blocks. Participants performed the *same-side* block first. In this block, a black heart was presented on the left or right side of the center fixation and the participants were asked to press the left or right button of a mouse corresponding to the target location (i.e., pressed the left button for a target on the left side). Participants then performed the *different-side* block. In this block, a black flower was presented on the left or right side of the center fixation and the participants were asked to press the opposite side of the target location (i.e., pressed the left button for a target on the right side). In the third block, a heart or a flower was randomly presented on the left or right side of the center fixation and the participants were required to follow the target-specific rule. They should adopt the same-side rule when a heart was presented and adopt the different-side rule when a flower was shown. The ability to swiftly switch between different rules reflects cognitive flexibility.

#### Procedure

Participants entered a room in which a 400 m × 500 m rectangle was marked with colored tape and a desk was situated in the center of the rectangle. Participants were invited to sit by the desk with the experimenter while listening to the purpose of the study and signing the consent form. Participants were told that the experiment was to investigate how context may influence problem solving and they were asked to read the instructions of the AUT while sitting. Participants in the rectangular-walking and free-walking groups then walked along the rectangular path or freely inside the rectangle, respectively, for 2 min while contemplating the solutions to the assignment. Participants in the free-generation and random-experienced groups were paired so that one was randomly assigned to generate a free walking path with a laser pointer while the other followed the path. Participants then wrote their AUT responses within 10 min. If participants finished the task before the time limit, they were encouraged to continue. Participants then filled the CAP instrument and performed the two cognitive tasks.

### Results and Discussion

#### Creativity Assessment Packet (CAP)

**Table 1** shows the CAP score of each group. There were no significant differences among the four groups [*F*(3,60) = 0.28, *p* = 0.840, η_p_^2^ = 0.014]. Participants did not differ in creativity traits.

**Table 1:** Mean performance and standard errors (*SE*, in parentheses) of the creativity assessment packet (CAP), two-alternative forced choice (2AFC task), and hearts and flowers task in Experiment 1.

	CAP	2AFC Accuracy	2AFC RT (ms)	Switching cost 1 (ms)	Switching cost 2 (ms)
Rectangular-walking group	57.50 (1.45)	0.99 (<0.01)	408 (18)	240 (17)	201 (18)
Free-generation group	57.75 (1.92)	0.99 (<0.01)	405 (15)	242 (29)	222 (16)
Random-experienced group	57.25 (1.87)	0.99 (<0.01)	414 (17)	245 (18)	176 (16)
Free-walking group	59.31 (1.75)	1.00 (<0.01)	406 (15)	248 (20)	201 (18)

*Switching cost 1 and cost 2 are the calculated by subtracting the RTs in the same-side block and opposite-side block from the RT in the mixed-block, respectively.*

#### 2AFC Task

Accuracy (see **Table 1**) was at the ceiling for all groups (99%), and there was no significant difference among groups (*p* > 0.80). The reaction times (RTs) of correct judgments were analyzed, excluding trials with RTs shorter than 70 ms or longer than 3000 ms. There was also no significant difference in RTs among groups (*p* > 0.90). Participants did not differ on general processing speed.

#### Hearts and Flowers Task

Accuracy was at the ceiling for all three blocks, and there was no significant difference in RTs among groups in each block (*p* > 0.30). The switching cost was calculated by subtracting the RTs of the same-side block and the opposite-side block from the RTs in the mixed-rules block. There was no difference in the two switching cost measures among groups (*p*s > 0.30).

#### The Alternative Uses Test (AUT)

Three independent raters who were naïve to the study purpose scored participants’ responses on the three components of divergent thinking. Fluency was measured by the number of non-repeated responses. Flexibility was evaluated by the number of categories of appropriate uses, which is the most stringent criterion for a use. Originality was defined by novel and appropriate responses compared to normed responses. The intra-class correlation coefficient (ICC) was employed to examine the degree of agreement by three raters on the same item. The ICC was quite high in each component of creativity. The ICC for fluency was 0.997 (95% confidence interval: 0.996–0.998, *F* = 1048.60, *p* < 0.001), the ICC for flexibility was 0.918 (95% confidence interval: 0.879–0.946, *F* = 34.42, *p* < 0.001), the ICC for originality was 0.942 (95% confidence interval: 0.914–0.963, *F* = 50.12, *p* < 0.001), all of which indicate extremely high inter-rater reliability. Scores were averaged across the three raters.

**FIGURE 1:**
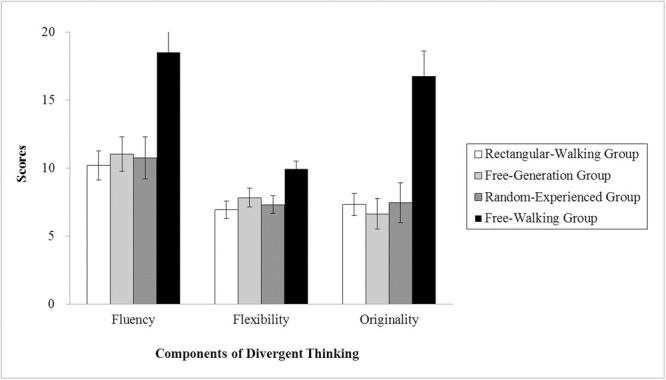
**Alternate uses test (AUT) performance in each group in Experiment 1.** Error bars represent *SE*s.

**Figure 1** shows the average scores among the four groups. Scores on the three components of divergent thinking were treated as three dependent measures, and a MANOVA was conducted using the groups (free-walking group, rectangular-walking group, free-generation group, random-experienced group) as a between-subject factor. An alpha level of 0.05 was used for all statistical tests. The results showed significant effects of groups among three components, *F*(3,60) = 8.44, *p* < 0.001, η_p_^2^ = 0.30 for fluency, *F*(3,60) = 4.30, *p* = 0.008, η_p_^2^ = 0.18 for flexibility, *F*(3,60) = 12.35, *p* < 0.001, η_p_^2^ = 0.38 for originality. The Scheffe test for the three components of creativity showed that the participants in the free-walking group performed better than the rectangular-walking group (*p* = 0.001), free-generation group (*p* = 0.003), and random-experienced group (*p* = 0.002) in fluency. Participants in the free-walking group performed better than the rectangular-walking group (*p* = 0.019) and the random-experienced group (*p* = 0.049) in flexibility; the free-walking group did not do better than participants in the free-generation condition (*p* = 0.16). Participants in the free-walking group performed better than the rectangular-walking group (*p* < 0.001), the free-generation group (*p* < 0.001), and the random-experienced group (*p* < 0.001) in originality. Compared with the rectangular-walking group, the effect of free walking on fluency (*r* = 0.62), flexibility (*r* = 0.52) and originality was strong (*r* = 0.64).

The results showed that bidirectional links between sensorimotor and metaphorical abstract concepts are critical for benefitting divergent thinking. Neither proprioceptive-motor kinematics alone in the random-experienced condition nor metaphorical abstract concepts *per se* in the free-generation condition could produce the benefits. Supporting the SSM model, integration of sensorimotor and conceptual components is critical for effective embodied cognition. More importantly, we observed benefits in all three components of divergent thinking. Thus, the null results of fluency and flexibility observed in Leung et al.’s (2012) experiment with young participants may have resulted from task insensitivity to the changes in these two measures.

One might question that the benefit of free walking arises from social signaling. Because participants were required to walk freely inside the box, they may have been primed to think outside the box without any constraints. By contrast, participants in the rectangular-walking condition were signaled to be rigid in thinking. We argue that social signaling is not a confounding factor for causing the benefits. If social signaling is the reason for observing the benefits, we should also observe benefits in the free-generation condition because these participants were also primed to think outside the box. Another concern is the social elements in pairing participants between the free-generation and random-experienced conditions. Although paired participants did not engage in any interaction, the paring itself may have caused potentially confounding effects. This concern is unlikely to explain the null results compared with the control group unless the pairing counteracts the benefits of metaphorical concepts or the benefits of sensorimotor execution. Social elements may not be a key factor for effective intervention because being more talkative to an experimenter while walking was not what produced benefits to divergent thinking (Oppezzo and Schwartz, 2014). A third possibility for observing the results is that walking provides an opportunity for mind-wandering or incubation to facilitate creative problem solving. Baird et al. (2012) showed that engaging in simple external tasks facilitates creative problem solving via promoting mind wandering. If mind wandering plays an important role, we would expect that participants in the rectangular-walking and free-generation conditions wandered more often that the participants in the random-experienced condition. In the former two conditions, the task was undemanding, following a pre-determined path or generating free paths without walking. In the latter condition, participants must pay attention to the paths generated by their partners and followed the paths. The results did not show any differences among these three groups.

Notably, the embodied experiences did not enhance performance on processing speed (2AFC task) and switching ability (the hearts and flowers task). It is likely that the cognitive measures reached the ceiling so that they were insensitive to changes in cognitive functions. This explanation applies only to the measure of processing speed because switch costs in reaction times were large. Moreover, it has been shown that the effect of training on cognitive functions is restricted to the trained tasks or tasks that share similar cognitive processes using similar materials (see Shipstead et al., 2012 for a review). Training is also effective after practicing trained activities for weeks. Training for juggling, for example, emphasizes hand-eye coordination. For such an activity, it could take from 6 weeks (Scholz et al., 2009) to 3 months (Draganski et al., 2004; Boyke et al., 2008) to induce changes in neural activity. For brisk walking to induce changes in neural networks related to cognitive functions, a period of 6 months is necessary (Colcombe et al., 2006). Two minutes of walking are likely insufficient to benefit cognitive functions.

## Experiment 2

The purpose of this experiment was to investigate whether free walking can also benefit divergent thinking in older adults. It has been shown that scores on divergent thinking were positively related to the complexity in neural networks for older adults (Ueno et al., 2015). Elders with higher scores on divergent thinking showed activated rather than a loss of complexity in neural networks. However, it is unclear whether older adults can benefit from intervention programs that promote divergent thinking. Older adults did not show improvement after 8 weeks of activities (e.g., brainstorming, poetry) that promoted divergent thinking (Flood and Scharer, 2006). By contrast, episodic-specificity induction can enhance divergent thinking for older adults (Madore et al., 2016). One factor may have caused the inconsistent results. Although educational levels were unreported in the former study, approximately 57.9% of the participants reported “barely get by” as their economic status. By contrast, participants in the episodic-specificity induction study were highly educated (*M*_educationlevel_ = 15.65, *SD* = 2.17). Leon et al. (2014) showed that older participants with high education levels (*M*_educationlevel_ = 17.23) could produce more unique responses on non-timed-constrained tasks of verbal divergent thinking compared to younger participants (*M*_educationlevel_ = 14.27). It is possible that only older adults with a higher education may benefit from interventions for divergent thinking ability. We investigated whether older adults with low levels of education can benefit from free walking.

### Method

#### Participants

Thirty-two older adults volunteered for the present experiment. These adults were recruited from a community center and received monetary rewards for their participation. None of the participants reported having a diagnosis of Alzheimer disease or a related disorder, history of stroke, head injury, psychiatric illness, or drug abuse problem on the self-report health-screening questionnaire. All participants reported corrected-to-normal vision and they were naïve to the purpose of the experiment.

All participants were over 65 years old, and the mean age was 74.06 years old (*SD* = 6.22). The participants were randomly assigned to two different groups: the rectangular-walking group and the free-walking group (*n* = 16 in each group). **Table 2** summarizes the group characteristics. There was no group difference in age, educational level, scores on the Mini-Mental State Examination (MMSE; Folstein et al., 1975) that assesses global cognitive function, working memory capacity, and reasoning ability (all *p* > 0.40).

**Table 2:** Group means and standard deviations (in parentheses) of age, education in years, global cognitive function measured with Mini-Mental State Examination (MMSE), and working memory performance in Experiment 2.

	Rectangular-walking group	Free-walking group
Age	74.75 (7.11)	73.38 (5.32)
Education	5.94 (4.60)	5.69 (3.00)
MMSE	22.94 (1.77)	23.63 (2.85)
Forward digit span	10.75 (3.32)	10.88 (2.92)
Backward digit span	4.69 (2.39)	4.19 (2.04)

#### Design, Task, and Procedure

All aspects were the same as those used in Experiment 1, except that only the rectangular-walking and free-walking conditions were conducted. On the AUT, participants spoke the unusual uses while the experimenter recorded the responses.

### Results and Discussion

#### Creativity Assessment Packet (CAP)

There was no significant difference between the two groups (*p* > 0.20) on the CAP measure. The two groups did not differ in creativity traits (see **Table 3**).

**Table 3:** Mean performance and standard errors (in parentheses) of the creativity assessment packet (CAP), two-alternative forced choice (2AFC task), and hearts and flowers task in Experiment 2.

	CAP	2AFC Accuracy	2AFC RT (ms)	Switching cost 1 (ms)	Switching cost 2 (ms)
Rectangular-walking group	55.69 (1.53)	0.99 (0.01)	659 (33)	496 (53)	331 (41)
Free-walking group	58.38 (1.96)	0.99 (0.01)	601 (25)	504 (40)	365 (54)

*Switching cost 1 and cost 2 are the calculated by subtracting RTs in the same-side block and opposite-side block from RT in the mixed-block, respectively.*

#### 2AFC Task

Accuracy was at the ceiling for both groups (99%), and there was no significant difference between groups (*p* > 0.90). RTs of correct judgments were analyzed, excluding trials with RTs shorter than 70 ms or longer than 3000 ms. There was also no significant difference in RTs between groups (*p* > 0.10). The two groups did not differ in processing speed (see **Table 3**).

#### Hearts and Flowers Task

Accuracy was at the ceiling in all three blocks, and there was no significant difference in RTs between groups in each block (all *p* > 0.40). The switching cost was calculated by subtracting the RTs in the same-side block and the opposite-side block from the RTs in the mixed-rules block. There was no difference in the two switching cost measures between the two groups (*p*s > 0.60). The two groups showed comparable performance in cognitive flexibility (see **Table 3**).

#### Alternative Uses Test (AUT)

The ICC was employed to examine the degree of agreement by the three raters on the same item. The ICC was extremely high in each component of creativity. The ICC for fluency was 0.993 (95% confidence interval: 0.988–0.996, *F* = 444.16, *p* < 0.001); for flexibility, 0.937 (95% confidence interval: 0.890–0.966, *F* = 45.36, *p* < 0.001); and for originality, 0.931 (95% confidence interval: 0.880–0.963, *F* = 41.51, *p* < 0.001), which all indicate extremely high inter-rater reliability.

**FIGURE 2:**
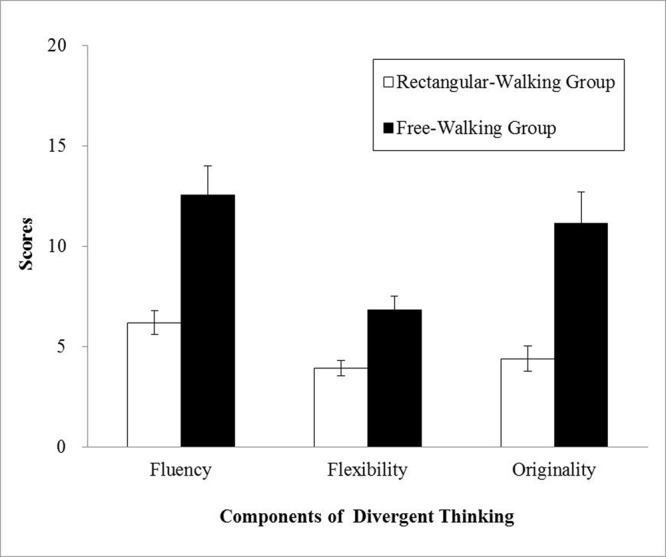
**Alternate uses test (AUT) performance of each group in Experiment 2.** Error bars represent *SE*s.

**Figure 2** shows the average scores of each group on the three measures of divergent thinking. Scores on the three components of creativity were treated as three dependent measures, and a MANOVA was conducted with the groups (free-walking group and rectangular-walking group) as a between-subject factor. The results showed a significant effect of groups among three components, *F*(1,30) = 16.77, *p* < 0.001, η_p_^2^ = 0.36 for fluency, *F*(1,30) = 12.74, *p* = 0.001, η_p_^2^ = 0.30 for flexibility, *F*(1,30) = 15.74, *p* < 0.001, η_p_^2^ = 0.34 for originality. Compared with the rectangular-walking group, the free-walking participants showed better divergent thinking with a large effect size (*r*s = 0.59, 0.53, 0.57 for fluency, flexibility, and originality, respectively).

### Cross-Experiment Comparison

The following two issues were of interest in this analysis: age effects on divergent thinking and whether free walking can enhance performance for older adults to a degree that is comparable to young adults in the rectangular-walking condition. Scores on the three components of creativity were then treated as three dependent measures, and a MANOVA was conducted with age (young adult compared with older adult) and walking condition (rectangular-walking compared with free-walking) as between-subject factors. The results showed that young adults earned higher scores on all three component measures of divergent thinking as follows: fluency [*F*(1,60) = 17.08, *p* < 0.001, η_p_^2^ = 0.22], flexibility [*F*(1,60) = 27.04, *p* < 0.001, η_p_^2^ = 0.31], and originality [*F*(1,60) = 10.56, *p* = 0.002, η_p_^2^ = 0.15]. Participants in the free-walking condition outperformed participants in the rectangular-walking condition for fluency [*F*(1,60) = 36.95, *p* < 0.001, η_p_^2^ = 0.38], flexibility [*F*(1,60) = 24.53, *p* < 0.001, η_p_^2^ = 0.29], and originality [*F*(1,60) = 37.75, *p* < 0.001, η_p_^2^ = 0.39]. The interaction did not reach significance for all three measures (*p*s > 0.30), showing that free walking is beneficial for both younger and older adults. To evaluate whether free walking can improve divergent thinking for older adults compared with young participants in the control condition, we conducted planned comparisons between the older free-walking group and the young rectangular-walking group. The results showed a significant difference in originality [*t*(30) = 2.15, *p* = 0.04, *d* = 0.76, *r* = 0.36] although the two groups did not differ on the other two measures (*p*s > 0.20). With free walking, older participants showed comparable or better performance by contrast to young adults who took a fixed walking path.

## General Discussion

This study had two objectives. First, we investigated the critical element that underlies the benefits of free walking on divergent thinking. Second, we examined whether free walking can benefit divergent thinking for older adults. The results from Experiment 1 showed that bidirectional links between proprioceptive-motor kinematics and metaphorical abstract concepts are critical for observing the benefits of free walking. The results of Experiment 2 showed that older adults can also benefit from free walking when generating unusual uses for a common object. More importantly, older adults in the free walking condition showed a comparable or better performance than young adults in the rectangular-walking control condition.

Supporting the hypothesis based on the SSM theory of embodied cognition, divergent thinking is enhanced only when the participants walked their own unconstrained, free paths. Activation of the concept without the sensorimotor element or vice versa could not produce the benefits to divergent thinking. It is plausible that our manipulation failed to activate the metaphorical concepts in the free-generation condition and to produce fluid movement in the random-experienced condition. Nevertheless, the benefits shown in the free-walking condition are extensive for both younger and older participants. An interesting finding that requires further investigation is the non-significant difference observed in Experiment 1 between the free-walking and free-generation groups on the flexibility measure. Although participants in the free-generation condition did not significantly outperform participants in the rectangular-walking condition, this null result suggests that activation of a metaphorical concept may enhance flexibility in divergent thinking.

To our knowledge, this is the first study that demonstrates the benefits of embodied cognition for older adults using the objective measures of divergent thinking. A previous study has shown that 4-week acting interventions may improve word recall and problem solving for older adults (Noice et al., 2004). Embodied cognition in acting includes multiple elements, including the multiple cognitive processes engaged in acting (e.g., mental simulation and deep processing of materials with meanings) in addition to affective and motoric elements (Noice and Noice, 2006). It is unknown which element is essential for acting to improve cognition. The essential element in free walking to benefit divergent thinking is the bidirectional links between conceptual representations and sensorimotor elements. In addition to art programs such as music, theater, dance, and design that promote healthy aging (Hanna et al., 2015), older adults could engage in free walking to improve their divergent thinking ability for creative problem solving in everyday life.

Compared with other art forms such as dance that nurture creative processes by embodiment, free walking can be conducted without any instruction or coaching. Over a short period of time (2 min in this study), this walking exercise improves divergent thinking. More importantly, we identified benefits for both highly educated young adults and less educated older participants. Note that the aging effect on divergent thinking is significant. Given the same condition (rectangular or free walking), older participants performed worse than younger participants. This finding is consistent with previous results (Alspaugh and Birren, 1977; Ruth and Birren, 1985; McCrae et al., 1987; Reese et al., 2001) but conflicts with the findings that age does not impair divergent thinking (Palmiero et al., 2014; Massimiliano, 2015; Madore et al., 2016) or that older adults performed better than younger adults on an AUT (Leon et al., 2014). Two factors may underlie age-related differences in the current study. First, we used a timed AUT. Although participants had 12 min to generate their responses, older adults, slower in processing speed, may not be able to generate as many unusual uses as young adults. Second, older participants were less educated than younger participants. Less educated older adults performed worse than highly educated young adults on divergent thinking under time constraints. However, free walking can enhance divergent thinking for less educated older adults. Importantly, older participants in the free-walking condition did not perform worse than younger participants in the rectangular-walking control condition. Older participants in the free-walking condition even outperformed young participants in the control condition on the originality measure. Free paths without angular corners at one’s own pace may be the important elements for the metaphors to influence divergent thinking, as walking indoors on a treadmill with a comfortable, self-selected pace or walking outdoors at one’s natural gait enhanced divergent thinking in young adults (Oppezzo and Schwartz, 2014). Older adults can take free walks at any time indoors or outdoors to generate novel and useful solutions for the problems they face. When running errands, older adults may adopt various routes rather than taking the same route to reduce their mental fixation. Through bodily movements that activate metaphorical concepts, older adults can improve their divergent thinking. With heightened ability, the loss of complexity in the neural networks may be reduced (Ueno et al., 2015), and self-efficacy may be strengthened for successful aging.

## Author Contributions

Y-YY and C-YK developed the study concept and design. Data was collected and analyzed by C-YK. Both authors interpreted the results. C-YK drafted the manuscript under the supervision of Y-YY, and Y-YY provided critical revisions. All authors approved the final version of the manuscript for submission.

## Conflict of Interest Statement

The authors declare that the research was conducted in the absence of any commercial or financial relationships that could be construed as a potential conflict of interest.

The reviewer AA and handling Editor declared their shared affiliation, and the handling Editor states that the process nevertheless met the standards of a fair and objective review.
